# Mental health variables affecting Quality of Life (QOL) among healthcare workers during the COVID-19 pandemic in Jazan City, Saudi Arabia

**DOI:** 10.3389/fpsyg.2024.1453494

**Published:** 2024-12-04

**Authors:** Amal Jaber Alfaifi, Ahmed Yahia Abdaly, Sultan Musa Alallah, Mohammad Zaino, Maged El-Setouhy

**Affiliations:** ^1^Department of Family Medicine, Jazan Health Cluster, Ministry of Health, Jazan, Saudi Arabia; ^2^Prince Mohammed bin Nasser Hospital, Jazan Health Cluster, Ministry of Health, Jazan, Saudi Arabia; ^3^Faculty of Nursing and Health Science, Physical Therapy Department, Jazan University, Jazan, Saudi Arabia; ^4^Department of Family and Community Medicine, Faculty of Medicine, Jazan University, Jazan, Saudi Arabia; ^5^Department of Community Environmental and Occupational Medicine, Faculty of Medicine, Ain Shams University, Cairo, Egypt

**Keywords:** quality of life, healthcare worker, COVID-19, Saudi Arabia, mental health

## Abstract

**Background:**

Health workers directly involved in the diagnosis, treatment, and care of patients with COVID-19 are at risk of developing mental health symptoms.

**Objective:**

The study aimed to assess the quality of life (QoL) of healthcare workers at Prince Mohammed bin Nasser Tertiary Hospital in Jazan during the COVID-19 pandemic, with a focus on the relationship between QoL and stress, anxiety, and depression. Pandemic.

**Methods:**

This was a cross-sectional study conducted among healthcare workers at Prince Mohammed bin Nasser Hospital in the Jazan. The study included a sample of 352 healthcare workers. Data was collected through a self-administered questionnaire pertaining to sociodemographic characteristics and the 21-item Depression Anxiety Stress Scale questionnaire, SF-36, for QoL. Descriptive statistics, frequencies, and percentages were used. A chi-squared test was performed to compare categorical data. A one-way ANOVA was performed to compare the effect of disorder variables on QoL. Multiple linear regression analyses were carried out to discern the differences between the different groups of participants in QoL measures.

**Results:**

Our results showed a poor QoL among those with a chronic disease (*p* = 0.002), who worked in the COVID-19 department (*p* = 0.030) and those who experienced the death of relatives or friends due to COVID-19 (*p* = 0.003).

**Conclusion:**

Healthcare workers, particularly those with chronic diseases or who had lost relatives to COVID-19, exhibited significantly lower QoL levels, especially those working directly in COVID-19 departments.

## Introduction

COVID-19, caused by the SARS-CoV-2 virus, was first identified in Wuhan, China, in late 2019 before rapidly escalating to a global pandemic (Liu et al., 2020; Juengling et al., 2020; Zangrillo et al., 2020). The rapid spread of the virus, transmitted primarily by human-to-human contact (Tfi et al., 2020; Mac Donald and Hsu, 2021; Karia et al., 2020) drove the World Health Organization to classify it as a pandemic in March 2020 (Cucinotta and Vanelli, 2020; Union et al., 2022; Res et al., 2018). To mitigate the spread of the virus, public health measures such as social distancing (Sun et al., 2022) quarantine and isolation were implemented (Oeltmann et al., 2023; Ayouni et al., 2021; World Health Organization, 2022). This would be the reason that the effects of this pandemic were not limited to physical health (Abbas et al., 2023; Su and Zhou, 2023) but also affected psychological (Fioravanti et al., 2020; Adorjan, 2023) and social well-being (King et al., 2023; Shevchenko et al., 2023; Vilar-Compte et al., 2022) including the safety of surrounding environments. Prevalence of depression, anxiety, and stress among healthcare workers was 29.4, 44.9, and 31.8%, respectively. Moreover, 90.3% of the healthcare workers had impaired physical components, and 156 (39.8%) healthcare workers had impaired mental components of the QoL (Syamlan et al., 2022). A recent study in Italy used a Lime survey platform to measure multiple forms of well-being, including mental health which was measured by Health Questionnaire (GHQ-12), which uses a 12-item scale, measures mental health and the severity of mental problems. Burnout measured by Maslach Burnout Inventory (MBI) measures perceived burnout and is organized into three subscales: emotional burnout (9 items), depersonalization (5 items), and realization (8 items). Compassion fatigue measured by the Compassion Satisfaction and Fatigue (ProQOL 5) scale measures feelings and negative and positive effects of those who help people in painful situations. The satisfaction of basic work-related needs measured by the Work-related Based Need Satisfaction (WrBNS) Scale and perceived support from friends and family. Among 340 healthcare worker participant, the study found a gradual decline in well-being due to stress, lead to psychological distress which appeared in the form of burnout (Rania et al., 2023). A study done in Egypt assessed anxiety related to COVID-19 infection, which is associated with QoL. It found that 28% of healthcare workers experienced health worries about the COVID-19 virus. Health anxiety in response to the COVID-19 virus was inversely associated with all domains of QoL in healthcare workers (Kandula, 2021). Another Egyptian study among 300 nurses in primary healthcare centers simple random sampling technique used to select sample 166 nurse from urban and 134 from rural health units’ QoL measured by the QoL scale of the World Health Organization. The result of study revealed negative consequences of COVID-19 on nurses’ physical, psychological, social, and environmental QoL. Moreover, most nurses experienced a low total QoL (80%), while only 20% had a high QoL (Mohamed et al., 2023). Concerning the health-related QoL among Jordan physicians during the COVID-19 pandemic, a cross-sectional study using a multiple-scale assessment included health-related QoL measured by the 12-item Short Form Health Survey, a neck disability index and a DASS-21. The DASS-21 found low QoL among physicians in Jordan during the COVID-19 pandemic (Almhdawi et al., 2022). A cross-sectional study targeted 19 Arab countries using the World Health Organization QoL-BREF instrument. The survey included 3,170 healthcare workers and found a large proportion of the Arab healthcare workers had an overall poor QoL (Fiidow et al., 2022). In Jazan City, 491 healthcare workers participated in a cross-sectional study in five secondary hospitals. The results reported symptoms of burnout symptom and a low level of QoL; 213 from the study sample reported arriving home late from work during the COVID-19 pandemic (Mahfouz et al., 2023).

Although tertiary hospital were dealing with complicated and urgent cases referred from other hospitals, no available data about mental health and quality of life among healthcare workers in those hospitals in Saudi Arabia during COVID-19 pandemic.

This study aimed to assess the quality of life (QoL) of healthcare workers at Prince Mohammed bin Nasser Tertiary Hospital in Jazan during the COVID-19 pandemic, with a focus on the relationship between QoL and stress, anxiety, and depression.

## Methodology

### Study design, setting, and participants

We conducted a cross-sectional study among healthcare workers in the Jazan region of Saudi Arabia. Jazan is located in the Southwest of Saudi Arabia and is adjacent to Yemen. It has a population of 1.5 million people. The study was conducted between December 2021 and April 2022 at Prince Mohammed bin Nasser Hospital, one of the region’s two tertiary hospitals. The participants were healthcare workers who spoke English and were available during the data collection.

### Sampling strategy

Our sampling strategies were meticulously calculated to ensure the reliability of our findings. The minimum required sample size was determined using the formula, *n* = ZxP2 (1-P)/D2, where n (calculated sample size) = 345, Z (the 95% confidence level) = 1.96, *p* (assumed prevalence in the population) = 50%, and *d* = 0.05.

We initiated our data collection process after obtaining ethical approval from the Jazan Ministry of Health’s research ethics committee. To mitigate the risk of COVID-19 transmission to the data collectors, we utilized an electronic web-based questionnaire (Google form) in English. Those who could not comprehend English were excluded from the sample. Data was collected through emails and disseminated to the selected sample by the hospital director to all healthcare workers.

### Data collection tool

We used a self-administered questionnaire to collect our data. It included three parts. The first part had the demographic and personal characteristics that would be associated with depression, anxiety, and stress among healthcare workers during the COVID-19 outbreak. The second part included the validated English version of the DASS-21, indicating acceptable internal consistency with Cronbach’s alpha of 0.959 (Thiyagarajan et al., 2022). Each of the three DASS-21 s contained seven items. The depression scale assessed dysphoria, hopelessness, devaluation of life, self-deprecation, lack of interest/involvement, anhedonia, and inertia. The anxiety scale assessed autonomic arousal, skeletal muscle effects, situational anxiety, and the subjective experience of anxiety. The stress scale was made sensitive to levels of chronic non-specific arousal. It assessed difficulty in relaxing, nervous arousal, impatience, irritability, and over-reactiveness. Scores for depression, anxiety, and stress were calculated by summing the scores for the relevant items with cut-off scores for conventional severity labels (i.e., regular, moderate, severe; Lovibond and Lovibond, 1995).

The third part of the questionnaire assesses QoL using the SF-36 questionnaire, which is valid and reliable (Cronbach’s alpha >0.85; Brazier et al., 1992). The scoring two-step process. First, pre-coded numeric values are recoded per the scoring. Note that all items are scored so that a high score defines a more favorable health state. In addition, each item is scored on a 0 to 100 range so that the lowest and highest possible scores are 0 and 100, respectively. Scores represent the percentage of the total possible score achieved. In step 2, items in the same scale are averaged together to create the eight scale scores: physical functioning, social functioning, role limitations (physical problems), role limitations (emotional problems), pain, mental health, vitality, and general health perception (Hays, 2017).

A pilot study involving 30 healthcare workers who were not included in the survey was conducted to make sure the questions and scale items were clear and understood and to determine how long it would take to complete the questionnaire.

### Statistical analysis

The study included a sample of 352 healthcare workers. Data was collected, coded, entered, and analyzed using Statistical Product and Service Solutions version 27 (IBM Corp., New York, NY, United States, 2019). Descriptive statistics, frequencies, and percentages were used for the categorical data to show the differences in percentages in sociodemographic characteristics regarding the QoL variable. A chi-squared test was performed to compare categorical data. A one-way ANOVA was used to compare the effect of disorder variables on QoL. A *post-hoc* test was conducted when the overall impact was significant to detect which disorder groups were responsible for that considerable effect.

Multiple linear regression analyses were carried out to determine the differences between the different groups of participants in QoL measures. The value *p* ≤ 0.05 was used to indicate a statistically significant result.

### Ethical consideration

The study was reviewed and approved by the IRB committee of the Jazan Hospital (H-10-Z-068) and the Ministry of Health in Saudi Arabia (IRB number 2191). Data (2-12-2021) Signed informed consent was obtained from all participants, and confidentiality and privacy were ensured.

## Results

**Figure 1:**
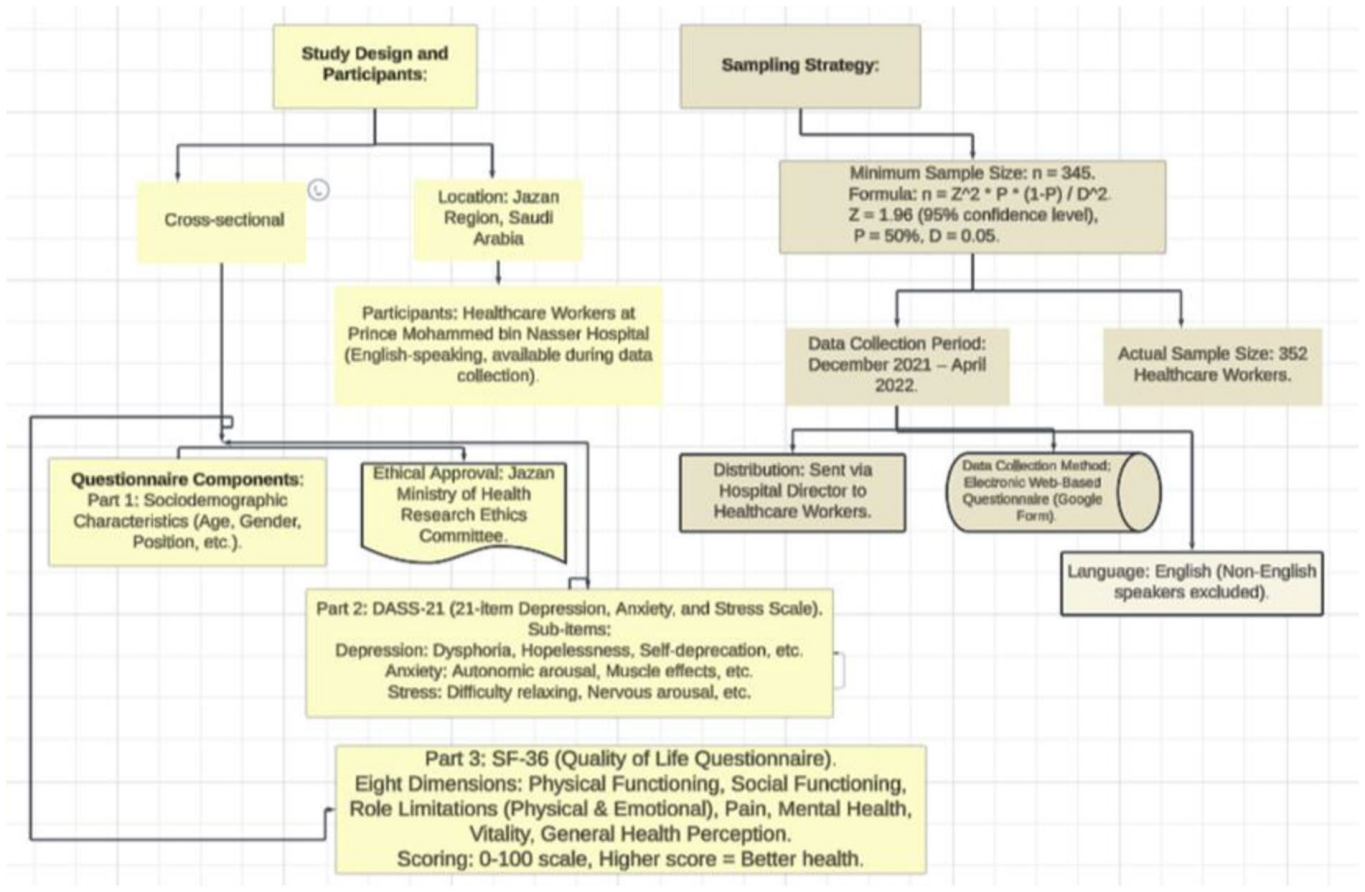
Flowchart: cross-sectional study of mental health and quality of life among healthcare workers in Jazan.

The main sociodemographic characteristics of the sample are shown in Table 1. The average age of the sample was 38.5 years. At the time of the interview, a third of the sample were married (Figure 1).

**Table 1:** Sociodemographic characteristics of the study group.

Variables	Total (*N* = 352)
Age	n (%)
20–29	275 (78.1)
30 and above	77 (21.9)
Sex	
Male	91 (25.9)
Female	261 (74.1)
Marital status	
Single	236 (67.0)
Married	116 (33.0)
Nationality	
Saudi	337 (95.7)
Others	15 (4.3)
Job title	
Physician	44 (12.5)
Applied medical science	229 (65.1)
Administrator	79 (22.4)
Years of working experience	
Less than 5 year	299 (84.9)
From 5 to 9 years	26 (7.4)
10 year and above	27 (7.7)
Having any chronic disease?	
Yes	35 (9.9)
No	317 (90.1)
Working in COVID-19 designated hospital?	
Yes	260 (73.9)
No	92 (26.1)
Death of families, relatives or friends due to COVID19?	
Yes	89 (25.3)
No	263 (74.7)
Having sufficient personal protective equipment?	
Yes	337 (95.7)
No	15 (4.3)

There were 275 (78.1%) participants aged 20–29 years, while 77 (21.9%) were aged 30 years and above. A gender majority of 261 (74.1%) were females, 337 (95.7%) were Saudi, and 236 (67%) were single. The majority of the studied sample was from applied medical science, representing 229 (65.1%), including those in lab work, radiography, nursing, social work, health education, public health, nutrition, physiotherapy, and medical information, with 44 (12.5%) physicians and 79 (22.4%) administrators. Looking at how long they worked in healthcare, 299 (84.9%) reported fewer than 5 years, and 27 (7.7%) reported more than 10 years. Thirty-five (9.9%) of the studied sample had a chronic disease, and most of the studied sample, 260 (73.9%), worked in a COVID-19-designated hospital. Eighty-nine (25.3%) had relatives die from COVID-19, and 337 (95.7%) had sufficient personal protective equipment.

The SF-36 Quality of Life (QoL) questionnaire was employed to evaluate the participants’ QoL levels. The scores were categorized into four levels: 0 (poor QoL), 25, 50, and 75 (favorable QoL). Participants working in COVID-19-designated hospitals and those who experienced the death of a family member or friend due to COVID-19 had significantly lower QoL scores (*p* ≤ 0.05), underscoring the profound impact of these factors on mental health (Table 2).

**Table 2:** Quality of life score in relation to sociodemographic characteristics among the studied sample: total (*N* = 352).

Sociodemographic characteristic	0	25	50	75	*p* value
Age (years)					0.444
20-29 (*N* = 275)	65 (23.6%)	71(25.8%)	67(24.4%)	72(26.2%)	
30 and above (*N* = 77)	23 (29.9%)	16 (20.8%)	22 (28.6%)	16 (20.8%)	
Sex					0.514
Male (*N* = 91)	19 (20.9%)	27 (29.7%)	24 (26.4%)	21 (23.1%)	
Female (*N* = 261)	69 (24.4%)	60 (23.0%)	65 (24.9%)	67 (25,7%)	
Marital status					0.265
Single (*N* = 236)	58 (24.6%)	61 (25.8)	53 (22.5%)	64 (27.1%)	
Married (*N* = 116)	30 (25.9%)	26 (22.4%)	36 (31.0%)	24 (20.7%)	
Nationality					0.869
Saudi (*N* = 337)	85 (25.2%)	84 (24.9%)	84 (24.9%)	84 (24.9%)	
Others (*N* = 15)	3 (20.0%)	3 (20.0%)	5 (33.3%)	4 (26.7%)	
Job title					0.295
Physician (*N* = 44)	10 (22.7%)	13 (29.5%)	12 (27.3%)	9 (20.5%)	
Applied medical science (*N* = 229)	61 (26.6%)	61 (26.6%)	51 (22.3%)	56 (24.5%)	
Administrator (*N* = 79)	17 (21.5%)	13 (16.5%)	26 (32.9%)	23 (29.1%)	
Years of working experience					0.536
Less than 5 years (*N* = 299)	74 (24.7%)	75 (25.1%)	72 (24.1%)	78 (26.1%)	
From 5–9 years (*N* = 26)	6 (23.1%)	5 (19.2%)	11 (42.3%)	4 (15.4%)	
10 years and above (*N* = 27)	8 (29.6%)	7 (25.9%)	6 (22.2%)	6 (22.2%)	
Having chronic disease					**0.002**
Yes (*N* = 35)	18 (51.4%)	5 (14.3%)	6 (17.1%)	6 (17.1%)	
No (*N* = 317)	70 (22.1%)	82 (25.9%)	83 (26.2%)	82 (25.9%)	
Working in covid19 designated hospital					**0.030**
Yes (*N* = 260)	55 (21.2%)	64 (24.6%)	71 (27.3%)	70 (26.9%)	
No (*N* = 92)	33 (35.9%)	23 (25.0%)	18 (19.6%)	18 (19.6%)	
Death of families, relatives or friends due to covid19					**0.003**
Yes (*N* = 89)	32 (36.0%)	27 (30.3%)	15 (16.9%)	15 (16.9%)	
No (*N* = 263)	56 (21.3%)	60 (22.8%)	74 (28.1%)	73 (27.8%)	
Having sufficient personal protective equipment					0.397
Yes (*N* = 337)	83 (24.6%)	83 (24.6%)	84 (24.9%)	87 (25.8%)	
No (*N* = 15)	5 (33.3%)	4 (26.7%)	5 (33.3%)	1 (6.7%)	

Bold values significant *p* < 0.05.

No significant associations were found between impaired QoL and factors such as age, gender, nationality, job title, working duration, or access to personal protective equipment.

The SF-36 questionnaire was distributed to 352 participants stress and normal, anxiety and normal, and depression and normal. A new disorder variable was constructed with four categories as follows: “normal,” “anxiety only,” “depression only,” and “depression and/or anxiety and/or stress.” A one-way ANOVA was performed to compare the effect of each of the subgroups’ physical functioning, role-physical, bodily pain, general health, mental health, role-emotional, social functioning, and vitality on the disorder variables categories “normal,” “anxiety only,” “depression only,” and “depression and/or anxiety and/or stress.”

The results in Table 3 revealed an overall statistically significant effect (*p*-value <0.001) in all subgroups (except physical functioning) on the disorder variable. A *post-hoc* Dunnett’s test for pairwise comparisons found that on the role-physical scale, participant with any of the mental problems “anxiety only,” “depression only,” and “depression and/or anxiety and/or stress” had significantly lower mean scores than the reference category normal (*p* = 0.032, 95% C.I. = [34.2, 71.3], *p* = 0.032, 95% C.I. = [22.2, 73.7], *p* = 0.008, 95% C.I. = [31, 69]), respectively.

**Table 3:** Average scores for the eight components (subgroups) of the SF-36 in relation to the variables of anxiety, depression, and stress.

Subgroups	Disorder	N	Mean ± SD	95% CI for Mean	ANOVA *p*-value	*Post-hoc p*-value
Physical functioning	Normal	302	67.3 ± 34.8	[63.4, 71.3]	0.074	
	Anxiety Only	18	53.1 ± 24.6	[40.8, 65.3]		0.215
	Depression Only	12	85.4 ± 17.5	[74.3, 96.5]		0.184
	Depression and / or Anxiety and / or Stress	20	70.3 ± 19.4	[61.2, 79.3]		0.974
Role-physical	Normal	302	75.5 ± 36.0	[71.4, 79.6]	0.000	
	Anxiety Only	18	52.8 ± 37.3	[34.2, 71.3]		**0.032**
	Depression Only	12	47.9 ± 40.5	[22.2, 73.7]		**0.032**
	Depression and / or Anxiety and / or Stress	20	50.0 ± 40.6	[31, 69]		**0.008**
Bodily pain	Normal	302	86.2 ± 22.9	[83.6, 88.8]	0.000	
	Anxiety Only	18	59.7 ± 23.3	[48.1, 71.3]		**0.000**
	Depression Only	12	67.3 ± 27.2	[50, 84.6]		**0.019**
	Depression and / or Anxiety and / or Stress	20	55.9 ± 28.7	[42.4, 69.3]		**0.000**
General health	Normal	302	69.5 ± 19.1	[67.3, 71.7]	0.000	
	Anxiety Only	18	50.1 ± 19.0	[40.7, 59.5]		**0.000**
	Depression Only	12	58.2 ± 18.7	[46.3, 70]		0.133
	Depression and / or Anxiety and / or Stress	20	41.4 ± 23.1	[30.5, 52.2]		**0.000**
Mental health	Normal	302	73.4 ± 21.9	[71, 75.9]	0.000	
	Anxiety Only	18	45.8 ± 19.0	[36.3, 55.2]		**0.000**
	Depression Only	12	45.7 ± 22.1	[31.6, 59.7]		**0.000**
	Depression and / or Anxiety and / or Stress	20	37.0 ± 19.3	[28, 46]		**0.000**
Role-emotional	Normal	302	76.0 ± 36.3	[71.9, 80.2]	0.000	
	Anxiety Only	18	61.1 ± 38.3	[42, 80.2]		0.261
	Depression Only	12	41.7 ± 45.2	[12.9, 70.4]		**0.005**
	Depression and / or Anxiety and / or Stress	20	40.0 ± 39.9	[21.3, 58.7]		**0.000**
Social functioning	Normal	302	78.9 ± 27.2	[75.8, 82]	0.000	
	Anxiety Only	18	58.8 ± 23.8	[46.9, 70.6]		**0.008**
	Depression Only	12	64.8 ± 33.5	[43.5, 86.1]		0.223
	Depression and / or Anxiety and / or Stress	20	46.6 ± 27.9	[33.6, 59.7]		**0.000**
Vitality	Normal	302	67.4 ± 22.6	[64.8, 69.9]	0.000	
	Anxiety Only	18	49.4 ± 17.6	[40.7, 58.2]		**0.003**
	Depression Only	12	30.8 ± 21.9	[16.9, 44.8]		**0.000**
	Depression and / or Anxiety and / or Stress	20	35.8 ± 18.9	[26.9, 44.6]		**0.000**

ANOVA *p*-value (Analysis of Variance): the overall *p*-value showing whether the difference in means between all groups is statistically significant. *Post-hoc p*-value (*Post hoc*, Dunnett-test): the pairwise *p*-value showing whether the difference in means between the reference normal group (no disorder) and each of the groups anxiety only, depression only, depression and/or anxiety and/or stress is statistically significant. Bold values significant *p* < 0.05.

On the bodily pain scale, participant with the mental disorder “anxiety only,” “depression only,” and “depression and/or anxiety and/or stress” had significantly lower mean scores compared to the category normal (*p* < 0.001, 95% C.I. = [48.1, 71.3], *p* = 0.019, 95% C.I. = [50, 84.6], *p* < 0.001, 95% C.I. = [42.4, 69.3]), respectively.

The presence of mental disorders “anxiety only” and “depression and/or anxiety and/or stress” implies a significantly lower average score in QoL among subgroups of general health compared to individual who have no mental disorder (*p* < 0.001, 95% C.I. [40.7, 59.5], *p* < 0.001, 95% C.I. = [30.5, 52.2]), respectively. Participants with “depression only” had lower scores on average than those in the normal category. However, the difference was not significant, *p* = 0.133.

On the mental health scale, the result shows that individual with mental disorders “anxiety only,” “depression only,” and “depression and/or anxiety and/or stress” had significantly lower mean scores compared to the category normal (*p* < 0.001, 95% C.I. = [36.3, 55.2], *p* < 0.001, 95% C.I. = [31.6, 59.7], *p* < 0.001, 95% C.I. = [28, 46]), respectively.

The results indicate that the mean scores on the QoL subscale role-emotional are significantly lower for the types of disorder “depression only” and “depression and/or anxiety and/or stress” (*p* = 0.005, 95% C.I. = [12.9, 70.4], *p* < 0.001, 95% C.I. = [21.3, 58.7]), respectively. Individuals with “anxiety only” had lower scores on average than those in the normal category; however, the difference was not significant (*p* = 0.261).

As shown in Table 3, the mean scores on the QoL subscale social functioning are significantly lower for the disorder “anxiety only” and “depression and/or anxiety and/or stress” (*p* = 0.008, 95% C.I. = [46.9, 70.6], *p* < 0.001, 95% C.I. = [33.6, 59.7]), respectively. People with “depression only” had lower scores on average than those in the normal category. However, the difference was not significant (*p* = 0.223).

On the vitality scale, the result displays that participant with mental disorders “anxiety only,” “depression only,” and “depression and/or anxiety and/or stress” had significantly lower mean scores compared to the category normal (*p* < 0.003, 95% C.I. = [40.7, 58.2], *p* < 0.001, 95% C.I. = [16.9, 44.8], *p* < 0.001, 95% C.I. = [26.9, 44.6]), respectively.

## Discussion

This study provides critical insights into the quality of life (QoL) of healthcare workers at a tertiary hospital in Jazan, Saudi Arabia, during the COVID-19 pandemic. Our findings revealed that healthcare workers with chronic diseases or who experienced the death of a relative or friend due to COVID-19 had significantly poorer QoL. This is consistent with a cross-sectional study conducted in 19 Arab countries, which found that healthcare workers who had a previous COVID-19 infection or lost relatives due to COVID-19 reported significantly lower QoL scores across (Fiidow et al., 2022). Similarly, a study conducted in seven Eastern African countries reported that healthcare workers with chronic diseases experienced lower QoL during the pandemic (Nizigiyimana et al., 2023).

A study in Bangladesh among healthcare workers in a tertiary hospital using the WHOQOL-BREF questionnaire also reported low QoL across the four domains: physical, psychological, social, and environmental. Factors such as being single, working long hours, and having a chronic disease were associated with poor QoL (Ei et al., 2023; Alfaifi et al., 2022). In Saudi Arabia, a study using the Kessler Psychological Distress Scale revealed that healthcare workers with conditions like hypertension and lower back pain were more vulnerable to psychological distress, which was exacerbated by the pandemic (Alfaraj et al., 2022).

Another Saudi Arabian study, which surveyed 4,920 healthcare workers across 13 regions, found a high prevalence of anxiety, particularly among unmarried nurses and those living with elderly relatives with chronic diseases (Alenazi et al., 2020). Anxiety, depression, and stress were also highly prevalent in a separate study conducted across different regions of Saudi Arabia, where healthcare workers related to someone who had died from COVID-19 reported higher anxiety levels (Almalki et al., 2021). A cross sectional study in Jazan city among quarantine healthcare workers during covid 19 pandemic also found association between anxiety and healthcare workers and comorbidity (Alfaifi et al., 2022).

Our findings are further supported by a systemic review of 19 studies involving 14,352 healthcare workers, which reported that professionals working directly with COVID-19 patients had lower QoL, particularly in relation to depression, anxiety, and stress (Spoorthy et al., 2020). So among healthcare workers during Covid-19 pandemic take in consideration those who have working direct with Covid-19 patients especially complicated cases.

A study conducted at the “Attikon” General University Hospital in Greece also found that anxiety and depression were negatively correlated with most subscales of QoL (Vamvakas et al., 2022).

In contrast to our findings, a multicenter study across five Saudi Arabian cities did not find any COVID-19-related variables significantly impacting QoL, despite using the WHOQOL-BREF instrument. Our study, however, demonstrated a clear link between lower QoL and COVID-19-related factors, such as working in COVID-19-designated departments and experiencing the loss of relatives to the virus (Pouralizadeh et al., 2020).

Regarding personal protective equipment (PPE), 95.7% of our participants reported having sufficient PPE. A study in New Delhi found that nearly 56% of healthcare workers were also satisfied with their PPE, but 10% reported shortages, which did not significantly affect their QoL. In our study, poor QoL was associated with healthcare workers in COVID-19 departments, in contrast to findings from the New Delhi study (Kaur et al., 2022). Another study from Turkey highlighted the impact of anxiety and the need for infection prevention control information on healthcare workers’ QoL during the pandemic (Mert et al., 2022).

Our results are consistent with a systemic review of psychological distress among healthcare workers during COVID-19, which found high levels of distress due to occupational factors, including reduced access to PPE (Arias-ulloa et al., 2023). Furthermore, a qualitative study conducted in Pakistan revealed that insufficient infrastructure and safety equipment had a significant physical and psychological impact on healthcare workers during the pandemic (Ma et al., 2023). During pandemic availability of personal protective equipment important to give the healthcare workers confidant to prevent transmission and reduce risk of mental illness and poor quality of life.

## Conclusion and recommendation

Healthcare workers, particularly those with chronic diseases or who had lost relatives to COVID-19, exhibited significantly lower QoL levels, especially those working directly in COVID-19 departments.

Occupational health clinics, work burnout clinics, and active screening for these psychological conditions could help reduce these mental disorders and improve QoL during a pandemic.

## Data Availability

The datasets presented in this study can be found in online repositories. The names of the repository/repositories and accession number(s) can be found in the article/supplementary material.
